# Differences in the mutation of the *p53* gene in exons 6 and 7 in cervical samples from HIV- and HPV-infected women

**DOI:** 10.1186/1750-9378-8-38

**Published:** 2013-10-07

**Authors:** Raquel P Souza, Fabrícia Gimenes, André LP de Abreu, Sheila C Rocha-Brischiliari, Maria DB de Carvalho, Érika C Ferreira, Marcelo G Bonini, Sandra M Pelloso, Marcia EL Consolaro

**Affiliations:** 1 Department of Clinical Analysis and Biomedicine, State University of Maringá, Av. Colombo 5790, Maringá 87020-900, Paraná, Brazil; 2 Department of Nursing, State University of Maringá, Av. Colombo 5790, Maringá, Paraná, 87020-900, Brazil; 3 Department of Medicine, State University of Maringá, Av. Colombo 5790, Maringá, Paraná, 87020-900, Brazil; 4 Department of Statistics, State University of Maringá, Av. Colombo 5790, Maringá, Paraná, 87020-900, Brazil; 5 Department of Pharmacology, University of Illinois, 909 S. Wolcott Ave, COMRB 3020, Chicago, IL 60612, USA

**Keywords:** HIV, HPV, Cervical lesions, *p53* gene, Mutations, Exons 5–8

## Abstract

**Background:**

Human *Papillomavirus* (HPV) infection is a serious problem for human immunodeficiency virus (HIV)-infected women, increases their risk of cervical lesions and cancer. In cervical carcinogenesis, mutations in the *p53* gene occur most frequently within exons 5–8. To our knowledge, no previous studies have analyzed mutations in exons 5–8 of the *p53* gene in HIV- and HPV-infected women. In our study, we verified these mutations in women with and without cervical abnormalities.

**Findings:**

The study included 160 women, divided into three groups: (1) 83 HPV- and HIV-infected women (HIV group); (2) 37 HPV-infected/HIV-uninfected (control group); and (3) 40 normal cytology/DNA-HPV negative/HIV-uninfected women (negative control *p53* reactions). HPV-DNA was detected using polymerase chain reaction (PCR) and genotyping by PCR-restriction fragment length polymorphism analysis. Using primers for exons 5–8, the mutation of the *p53* gene was verified by PCR-single strand conformational polymorphism. The total mutation of the *p53* gene in exons 5–8 was not significantly associated with the HIV and control groups. The mutations in exon 7 were the highest in the HIV group (43.8%) and in exon 6 in the control group (57.2%) (p = 0.0793) suggesting a tendency toward differential mutation in exon 7 in the HIV group.

**Conclusions:**

Our study provides preliminary evidence that the mutation in exon 7 might be an important differentiating factor for cervical carcinogenesis in HIV-infected women. This aspect deserves an additional cross-sectional and longitudinal study using a larger sample size with a higher number of High-grade squamous intraephitelial lesion (HSIL) to observe the evolution of cervical lesions.

## Findings

Human *Papillomavirus* (HPV) infection is a particularly difficult problem for human immunodeficiency virus (HIV)-infected women because they are more vulnerable to infection and less likely to clear the virus, which increases their risk of developing cervical lesions and cancer [1]. Specifically, there are differences in the prevalence, incidence, progression and regression of HPV-related cervical diseases in HIV-infected women compared to HIV-uninfected women. Moreover, in HIV-infected women, cervical cancer (CC) responds poorly to recommended therapies, behaves more aggressively, and in cases of recurrence, has a poorer prognosis [1,2]. The severe impact of HIV in relation to CC was demonstrated in a study that showed that HIV-positive women had an almost five fold greater chance of developing precancerous lesions than did HIV-uninfected women [3].

In cervical carcinogenesis, the integration of high-risk HPV (HR-HPV) into host-cell chromosomes is followed by the binding of HPV E6 and E7 oncoproteins with tumor suppressor proteins *p53* and *pRb*, respectively. This process results in impaired tumor suppressor gene function, involving DNA repair, decreased apoptosis, deregulation of key controls in cell proliferation, and eventual cell immortalization [4].

The *p53* tumor suppressor gene specifically inhibits cell cycle progression and promotes DNA repair and/or apoptosis; its inactivation is correlated with a critical step in the development of many human cancers. Inactivation may result from a number of events, including mutation of the *p53* gene (with or without associated allelic deletions) and binding of the *p53* gene to cellular or viral proteins, such as the HPV E6 oncoprotein [5]. *p53* mutations are mostly missense in nature and are located predominantly within the DNA-binding domain, where a single mutation is sufficient to cause loss of normal *p53* function [6]. Studies on the association between CC and the loss of *p53* function have yielded conflicting results [5,7,8].

Mutations in the *p53* gene occur most frequently within exons 5–8, which is the highly conserved DNA binding domain region [9]. Studies on *p53* that included all exons have suggested that mutations outside exons 5–8 are rare in tumors [10]. However, most studies on the involvement of the *p53* gene in cervical carcinogenesis are related to its protein expression by immunohisto/cytochemical or polymorphism in condon-72 [5,7]. To our knowledge, no previous studies have analyzed the mutations in exons 5–8 of the *p53* gene in HIV- and HPV-infected women. In our study, we verified these mutations in women with and without cervical abnormalities.

The study included cervical samples of 160 women who were divided into three groups: (1) 83 HPV- and HIV-infected (HIV group); (2) 37 HPV-infected/HIV-uninfected (control group); and (3) 40 cervical samples from women with normal cytology/DNA-HPV negative/HIV-uninfected that were only used as a negative control for the mutation of the *p53* gene exons (negative control *p*53 reactions). The women included in the study were enrolled in the Specialized Assistance Service (SAE) for sexually transmitted diseases (STD)/AIDS in Maringá, Brazil, between April 1, 2011, and October 30, 2011. In these samples, HPV-DNA was previously detected (data not shown) in the Clinical Cytology Laboratory at the State University of Maringá (UEM), Brazil, and stored at -80°C. Stored samples from HIV-infected and HIV-uninfected women, both with and without cervical lesions, were included in the study. The samples for 1 and 2 groups were also HPV-DNA positive.

Cervical and endocervical samples were collected using a cytobrush and an Ayre’s spatula, transferred to 1.5 ml tubes with 1.0 ml of sterile 0.9% NaCl solution, and stored at -80°C. Genomic DNA was extracted using the AxyPrep™ Body Fluid Viral DNA/RNA Miniprep Kit (AP-MN-Bf-VNA-50, Axygen, CA, USA) according to the manufacturer’s instructions. The quality and quantity of purified DNA were measured by spectrophotometry. HPV polymerase chain reaction (PCR) amplification for HPV was conducted using primers MY09 (5′CGTCCMAARGGAWACTGATC-3′) and MY11 (5′-GCMCAGGGWCATAAYAATGG-3′); co-amplification of the human β-globin gene was performed as an internal control using primers GH20 (5′-GAAGAGCCAAGGACAGGTAC-3′) and PC04 (5′-CAACTTCATCCACGTTCACC-3′) under the same conditions as for the HPV-PCR [11]. The cytological smears were prepared with a portion of the collected material and were reported according to the Bethesda System [12]: normal, without altered cells; atypical squamous cells of undetermined significance, cannot exclude high-grade squamous intraepithelial lesion (ASC-H); low-grade squamous intraepithelial lesion (LSIL); or high-grade squamous intraepithelial lesion (HSIL). Cases with a cytological diagnosis of HSIL were confirmed by histopathology.

For HPV genotyping of selected samples, a new HPV-PCR amplification was performed, which was followed by PCR-Restriction Fragment Length Polymorphism (PCR-RFLP) analysis using *Hpy*CH4V [13]. The mutations of the *p53 g*ene was verified by PCR-Single Strand Conformational Polymorphism (PCR-SSCP), which detects molecular changes in single-stranded DNA that cause changes in electrophoretic mobility [8]. The following primers were used: exon 5 (5′-TGTTCACTTGTGCCCTGACT-3′) / (5′-AGC AAT CAG TGA GGA ATC AG-3′), 310 bp; exon 6 (5′-TGGTTGCCCAGGGTCCCCAG-3′) / (5′-TGGAGGGCCACTGACAACCA--3′), 223 bp; exon 7 (5′-CTTGCCACAGGTCTCCCCAA-3′) / (5′-AGGGGTCAGCGGCAAGCAGA-3′), 248 bp; and exon 8 (5′-TTGGGAGTAGATGGAGCCT-3′) / (5′-AGAGGCAAGGAAAGGTGATA-3′), 213 bp. This is a simple technique which allows the identification of samples with type missense mutation, as well as correlation with the expression of mutated *p53*[14]. The women signed a consent form, and this study was approved by the Committee for Ethics in Research Involving Humans at the State University of Maringá (UEM)/Paraná, Brazil (No. 085/2011).

The statistical analysis was performed using STATISTICA 8.0 software, and all of the variables were expressed as absolute and relative frequencies. The rates of *p*53 exon mutations in the groups of women were compared using a non-parametric Z test. A p value < 0.05 was considered statistically significant.

On average, the HIV group was older than the control group (40.9 ± 11.23 *vs* 35.7 ± 10.57 years old; p *=* 0.0254). The majority of the HIV group showed excellent control of the HIV infection, as determined by the correct use of highly active antiretroviral therapy (HAART) (79.2%), current CD4+ T lymphocyte count > 350 cells/mm^3^ (73.6%) and current viral load < minimum limit (58.4%) or between the minimum limit and 100 copies/mL (38.8%).

The most common HR-HPV in the HIV group were HPV-51 and HPV-16 (n = 11/83, 13.5% each). Of these samples, a *p53* mutation occurred in only 1 sample of HPV-16 (9.1%) and in 6 samples of HPV-51 (54.5%). For the control group, HPV-16 (n = 6/37, 16.2%) and HPV-66 (n = 5/37, 13.5%) were the most common, but the *p53* mutation was observed in only 1 sample each (16.7% and 20.0%, respectively). HPV-58 was detected in 2 samples, and the mutation occurred in these 2 samples (100%). In regards to the cytological findings, the HIV group showed the following: 70 normal, 2 ASC-H, 10 LSIL and 1 HSIL. The findings in the control group were as follows: 21 normal, 6 ASC-H, 9 LSIL and 1 HSIL (Table 1).

**Table 1:** **Distribution of mutations in the *p53 *gene in HIV group and control group**

**Cytologic findings**	**Total samples**		***p53 *mutation**		**Total samples**		***p53 *mutation**		*p
	**N**	**%**	**N**	**%**	**N**	**%**	**N**	**%**	
Normal Cytology	70	84.3	12	17.1	21	56.8	4	19.0	0.4956
ASC-H	2	2.4	1	50.0	6	16.2	-		-
LSIL	10	12.1	2	20.0	9	24.3	3	33.3	0.3303
HSIL	1	12	1	100.0	1	2.7	-		-
TOTAL	83	100.0	16	19.3	37	100.0	7	18.9	0.4896

*HPV*, human papillomavirus; *HIV*, human immunodeficiency virus; cytologic findings (according to Bethesda System 2001): atypical squamous cells of undetermined significance, cannot exclude high-grade squamous intraepithelial lesion (ASC-H); low-grade squamous intraepithelial lesion (LSIL); high-grade squamous intraepithelial lesion (HSIL).

*p < 0.05 was considered significant.

**Figure 1:**
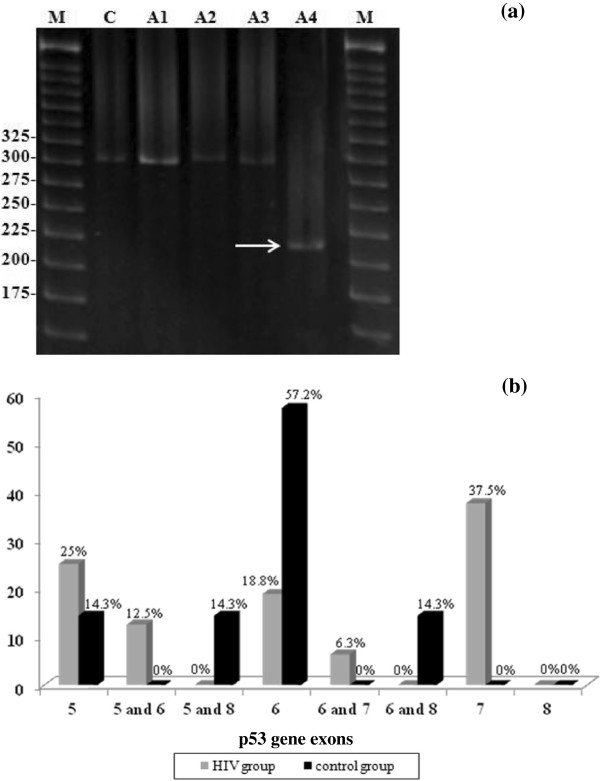
**Mutation in exon 6 of the *p53 *gene in the HIV group compared with the control group.** Panel **a**: Electrophoretic analysis of cervical *p53* gene mutations using PCR-Single Strand Conformational Polymorphism (PCR-SSCP) in an 8% polyacrylamide gel stained with ethidium bromide. Samples A1-A3, negative for *p53* gene mutations; A4, positive for *p53* gene mutation (arrow); C, negative control *p*53 reactions; M, 25 bp molecular weight marker. Panel **b**: The frequency of mutations in *p53* exons 5 to 8 in both groups. Exon 7 and exon 6 were the most mutated exons examined in the HIV group and in the control group, respectively.

Figure 1(a) shows a mutation in exon 6 as an example of abnormal bands of the *p53* gene. In total, 19.3% of the HIV group and 18.9% of the control group (p = 0.4896) showed mutations in the *p53* gene and all had HR-HPV. As previously described, no mutations occurred in any of the negative control *p*53 reactions [15] which is baseline condition this population. A mutation in exon 7 alone or together with a mutation in exon 6 was the highest in the HIV group (43.8%), and a mutation in exon 6 was highest in the control group (57.2%) (p = 0.0793) (Figure 1b). However, because the p value was close to being statistically significant, these data may suggest a tendency toward mutations in exon 7 in the HIV group.

In the HIV group, a mutation in exon 7 occurred at the highest frequency in normal cytology (31.3%) followed by LSIL and HSIL (6.3% for both). For the control group, a mutation in exon 6 was most common in both normal cytology and LSIL (28.6% each) (Table 1). There was no difference in mutation rates in normal cytology or lesions in both groups (p = 0.4956 and 0.3303, respectively; Table 1).

These findings suggested that the HPV infection appears to lead to a mutation in the *p53* gene in different exons in HIV-infected and HIV-uninfected women, i.e., mainly in exons 7 and 6, respectively. A point mutation at the splice donor site at the 3′ end of exon 7 of the human *p53* gene results in the retention of the intron 7 sequence in the mRNA, thereby inactivating the *p53* protein [16].

We also showed that the total mutation in *p53* gene exons 5–8 was not significantly associated with the HIV and control groups, and in women of both groups with normal cytology or different grades of cervical abnormalities, similar to those described for *p53* gene condon 72 polymorphism [7] and *p53* gene immunoexpression [5] in HIV-uninfected women. Interestingly, the samples from the HIV group with HPV-51 showed the highest rates of mutations in the *p53* gene which deserves further studies. Overall, higher rates of cervical HPV infection and CC can be partly related to a more specific mutated exon in HIV-infected patients.

Through its effect on CD4 cells and regulation of immune responses to a variety of antigens, HIV infection may attenuate the systemic immune response to HPV. It is speculated that if there is a low number of circulating HPV-specific memory cells, then HPV-specific immunity may be particularly vulnerable to the effects of HIV. Possible due to this HIV-infected women are more vulnerable to infection and less likely to clear the virus, which increases their risk of developing cervical lesions and cancer [1]. According to this hypothesis, HPV-specific immunity may not recover fully after immune response is restored, which may explain the relatively limited beneficial effect of HAART on HPV cervical infection and CC [17].

## Conclusions

In conclusion, we acknowledge that we did not study women with CC, and we studied few women with HSIL. However, our study cannot exclude the possibility that the mutations in the *p53* gene may act at later stages of carcinogenesis. Nevertheless, we believe that our study provides preliminary evidence that the mutation in exon 7 might be an important differentiating factor for cervical carcinogenesis in HIV-infected women. This aspect deserves an additional cross-sectional and longitudinal study using a larger sample size with a higher number of HSIL to observe the evolution of cervical lesions.

## Abbreviations

HPV: Human Papillomavirus; HIV: Human immunodeficiency virus; HR-HPV: High risk-HPV; p53: Related protein kinase; pRb: Phosphorylate the retinoblastoma; CC: Cervical cancer; SAE: Specialized assistance service; STD: Sexually transmitted diseases; AIDS: Acquired immuno-deficiency syndrome; PCR: Polymerase chain reaction; ASC-H: High-grade squamous intraepithelial lesion; LSIL: Low-grade squamous intraepithelial lesion; HSIL: High-grade squamous intraepithelial lesions; RFLP: Restriction fragment length polymorphisms; SSCP: Single strand conformational polymorphism; OpenEpi: Open Source Epidemiologic Statistics for Public Health; HARRT: Highly active antiretroviral therapy

## Competing interests

The authors declare that they have no competing interests.

## Authors’ contributions

All authors contribute to the manuscript. RPS, FG, ALPA and SCR-B searched the literature and manuscript preparation. SCR-B, MDBC and SMP collected the women biological samples. MELC, ALPA and FG wrote the manuscript. MELC, RPS, FG and ALPA participated in methodology design and execution. SCR-B, MDBC and SMP contribute to the statistical analysis and design of the study. MDBC, SMP and MELC had been involved in revising the manuscript critically for important intellectual content. MELC revised the final manuscript version, helped to provide information and suggestion. All the authors read and approved the final of the manuscript.
